# Mindfulness-Based Social Cognition Training (SocialMind) for People With Psychosis: A Feasibility Trial

**DOI:** 10.3389/fpsyt.2019.00299

**Published:** 2019-05-02

**Authors:** Roberto Mediavilla, Ainoa Muñoz-Sanjose, Beatriz Rodriguez-Vega, Carmen Bayon, Guillermo Lahera, Angela Palao, Maria Fe Bravo-Ortiz

**Affiliations:** ^1^Hospital La Paz Institute for Health Research (IdiPAZ), Madrid, Spain; ^2^School of Psychology, National University of Distance Education (UNED), Madrid, Spain; ^3^Department of Psychiatry, Clinical Psychology and Mental Health, La Paz University Hospital, Madrid, Spain; ^4^School of Medicine, Autonomous University of Madrid (UAM), Madrid, Spain; ^5^School of Medicine, University of Alcala (UAH), Madrid, Spain; ^6^Mental Health Networking Biomedical Research Centre (CIBERSAM), Madrid, Spain

**Keywords:** mindfulness, social cognition, psychosis, schizophrenia, feasibility, clinical trial, SocialMind

## Abstract

**Introduction:** Difficulties in social functioning are common among people with psychosis. Negative symptoms such as blunted affect or social withdrawal are often linked to these difficulties and worsen real-life outcomes. One important dimension associated with social functioning is social cognition, which refers to the psychological processes that are necessary to perceive, encode, store, retrieve, and regulate social information. Mindfulness-based interventions for people with psychosis are safe and effective in improving anxiety and depressive symptoms; however, no mindfulness-based interventions addressing social cognition have yet been developed.

**Method:** A pilot, single-arm, nonrandomized, noncontrolled feasibility trial is proposed. The main objectives are to assess the tolerability of mindfulness-based social cognition training (SocialMind) and to test the feasibility of a further randomized controlled trial.

**Results:** A final sample of 25 outpatients with schizophrenia spectrum disorders was included. Attrition rate was lower than usual for this population, and most participants completed the training. No adverse effects were identified in terms of hospitalizations, emergency room visits, dissociative and psychotic symptoms, or state of anxiety during the sessions.

**Conclusion:** This is the first implementation of SocialMind, which is the first mindfulness-based social cognition training. It is well tolerated by participants with schizophrenia spectrum disorders, and a further randomized controlled trial is proposed for people who have suffered their first episode of psychosis within the past 5 years.

**Clinical Trial Registration:** www.ClinicalTrials.gov, identifier NCT03434405.

## Introduction

People suffering from psychosis frequently find it difficult to establish or maintain relationships with others or to engage in community activities (1, 2). These deficits are present even in high-risk or prodromal states (3), strengthening the role of social functioning as a core therapy outcome (4, 5). Social cognition is defined as “the psychological processes that are involved in the perception, encoding, storage, retrieval, and regulation of information of other people and ourselves” (6). It is associated with community functioning, with estimated average correlations ranging from 0.31 to 0.48 (7), and its deficits can also be observed in the early stages of the psychotic process (8).

Antipsychotic medication improves positive and general psychiatric symptoms, but it has limited effect against negative syndrome, which consists of blunted affect, apathy, lack of spontaneity, and social withdrawal (9). These symptoms often respond to some psychological interventions, such as assertive community treatment, cognitive remediation, social skills training, or cognitive-behavioral therapy (10); hence, they are usually offered along with antipsychotic treatment (11). Many cognitive-behavioral interventions are now available for people with psychosis. They have evolved from targeting only emotional distress to focusing on psychotic symptoms (10), and have been shown to be effective in improving these symptoms (12) but unable to achieve changes in real-life outcomes (13). These psychotherapeutic approaches emphasize the effort the person needs to make to modify some thoughts, feelings, or behaviors that are harmful: a rationale that fits within an illness-centered framework. In contrast, third-wave cognitive-behavioral interventions are person-centered, emphasize contextual and experiential (rather than behavioral) changes, and explicitly target the functional dimension of psychological phenomena (14, p. 880).

Mindfulness-based interventions (MBIs) have gathered strong empirical support for both physical conditions, such as cancer, chronic pain, or cardiovascular disease (15), and mental health problems, such as anxiety, depression, addictive behavior, or schizophrenia spectrum disorders (SSDs) (16). MBI trainees learn attentional strategies that promote awareness of the present moment, as well as cultivate a curious nonjudgmental attitude towards their own experience. This modification of the attentional process fosters a different relationship with symptoms and is one of the active constituents for distress reduction and functional recovery (17, 18).

Proliferation of MBIs for people with psychosis have drawn attention to the adverse effects that may emerge, such as dissociative and anxiety symptoms or psychotic-like experiences (19). A recent systematic review and meta-analysis of randomized controlled trials (RCTs) concludes that MBIs are well tolerated by people with SSDs and can be implemented in addition to pharmacological treatment (20). Furthermore, mindfulness training might improve both negative (21) and depressive (22) symptoms.

SocialMind is a mindfulness-based social cognition training with the aim of improving social functioning of people who have suffered at least one episode of psychosis. It is a novel, complex intervention tailored for people with significant levels of distress; furthermore, professionals who implement the intervention need to be experienced both in the field of psychosis and in the teaching of mindfulness techniques. A first approach to SocialMind is thus required in order to explore whether it is safe and well tolerated, participants adhere to the training, and teachers comply with the intervention manual. A feasibility trial allows us to test the hypothesis that SocialMind is acceptable and tolerable for people with psychosis in terms of adverse effects, participant satisfaction, and attrition rates (23). Further, recruitment rates will provide some indirect evidence regarding the power to conduct a further RCT with parallel groups (SocialMind *versus* active comparator).

## Materials and Methods

### Study Design and Participants

It is a pilot, open-label, single-group, nonrandomized, noncontrolled feasibility trial. Participants were recruited from the Psychiatry, Clinical Psychology, and Mental Health Department of La Paz University Hospital in Madrid (Spain) during January and February 2018. All were engaged in either pharmacological treatment, psychosocial treatment, or both. People with a duration of psychosis of less than 5 years were not eligible, as they will be contacted to enroll in the upcoming RCT. The research was approved by the La Paz University Hospital Research Ethics Committee (identifier PI-3066) and registered under ClinicalTrials.gov, identifier NCT03434405.

Inclusion criteria were as follows:

1. Between 18 and 60 years of age,
2. Diagnosis of SSD according to the Diagnostic and Statistical Manual of Mental Disorders, 5th Edition (DSM-5),
3. A Clinical Global Impression (CGI) (24) score equal to or less than four (moderately ill), and
4. Signed informed consent.

Exclusion criteria were as follows:

1. First hospitalization, first visit to mental health services with positive symptoms, or first appearance of positive symptoms confirmed by an informant after January 2013;
2. Intellectual disability plus impaired function prior to disorder onset,
3. Generalized developmental disorder; and
4. Substance-related disorder (except for nicotine) according to DSM-5.

### Instruments

*Symptom Checklist 90—Revised (SCL-90-R)* (25). This instrument consists of a list of 90 symptoms from nine different domains of psychopathology (depression, anxiety, paranoia, psychoticism, etc). The individual has to rate how he or she experienced each symptom during the last week. Nine domain-specific scores are provided, as well as three general indexes. The Spanish version of SCL-90-R has high internal consistency (Cronbach’s alpha from 0.81 to 0.90) and good convergent and criterion validity, and it is very sensitive for detecting changes (26).

*Dissociative Experiences Scale II (DES-II)* (27). This scale explores the amount of time a person has a certain experience (i.e., the experience of driving somewhere and not recalling how one got there). There are 28 experiences to be scored from 0% to 100% of the time. The final score is an average percentage of time. The Spanish version has good internal consistency (Cronbach’s alpha = 0.91) and detects differences between people with schizophrenia and nonclinical samples (28).

*State-Trait Anxiety Inventory—State subscale (STAI-S)* (29). The individual has to rate his or her degree of anxiety at the moment of completing the scale. The score ranges from 0 to 60. The Spanish version (30) has high internal consistency for clinical samples (Cronbach’s alpha = 0.98) and good test–retest reliability (r = 0.93) (31); furthermore, scores are very sensitive to stressful environments (32).

*Positive and Negative Syndrome Scale (PANSS)* (33). This scale explores three main dimensions through a semistructured interview: positive syndrome (PANSS-P), negative syndrome (PANSS-N), and general psychopathology (GP). Values range from 1 (“absent”) to 7 (“extreme”), and final scores range from 7 to 49 for PANSS-P and PANSS-N and from 16 to 112 for GP. Subscales from the Spanish version are strongly associated with the original version (r = 0.92 for PANSS-P and r = 0.83 for PANSS-N), with item correlations ranging from r = 0.64 to r = 0.97 and high interrater reliability (r = 0.81) (34).

*Personal and Social Performance (PSP) scale* (35). This scale explores four domains of social functioning, namely, self-care, social relationships, social activities, and disruptive and aggressive behavior. The final score ranges from 0 to 100. The Spanish version of the PSP is reliable and has high internal consistency (Cronbach’s alpha = 0.87) and excellent test–retest reliability (r = 0.98); moreover, it comprises one single component that explains 73% of the variance in social functioning (36).

*Client Satisfaction Questionnaire (CSQ-8)* (37). This eight-item instrument explores how much a person is satisfied with different domains of healthcare services. A final score between 8 and 32 is provided. The Spanish version has levels of internal consistency similar to the original version (Cronbach’s alpha = 0.90); however, final scores appear to be influenced by job status and education level (38). It has been previously used in feasibility trials of MBIs for psychosis (39).

*SocialMind teachers’ checklist*. This instrument consists of two parts. The first is inspired by the assessment of protocol compliance in Social Cognition and Interaction Training (SCIT) (40). Raters must check if teachers adhere to the SocialMind manual and complete eight items that range from 0 to 2 points, with higher values indicating more adherence. The second part is the Mindfulness-Based Interventions: Teaching Assessment Criteria (MBI:TAC) (41), which comprises six domains that should be addressed in each session, such as embodiment of mindfulness, correct guidance, or holding the group environment. Teachers can obtain a score between 1 (“incompetent: absence of key features or highly inappropriate performance”) and 6 points (“advanced: excellent teaching practice, or very good even in the face of participant difficulties”). Assessments were made after checking video recordings of the sessions.

### Procedure

#### Assessment

The pre-intervention assessment was completed within 2 weeks prior to the start of SocialMind training. It consisted of a sociodemographic interview, followed by PANSS and PSP (clinical interview) and by SCL-90-R and DES-II (self-reported instruments). The post-intervention assessment was carried out within 2 weeks after the end of SocialMind training and included PANSS, PSP, SCL-90-R, DES-II, and CSQ-8. STAI-S was completed before and after each session. Raters were psychologists and intern psychiatrists who were not SocialMind teachers.

For the assessment of the teachers, they watched video recordings of sessions in which they had not participated. This ensured that raters were familiarized with the intervention and avoided self-rating.

#### Intervention

**Figure 1:**
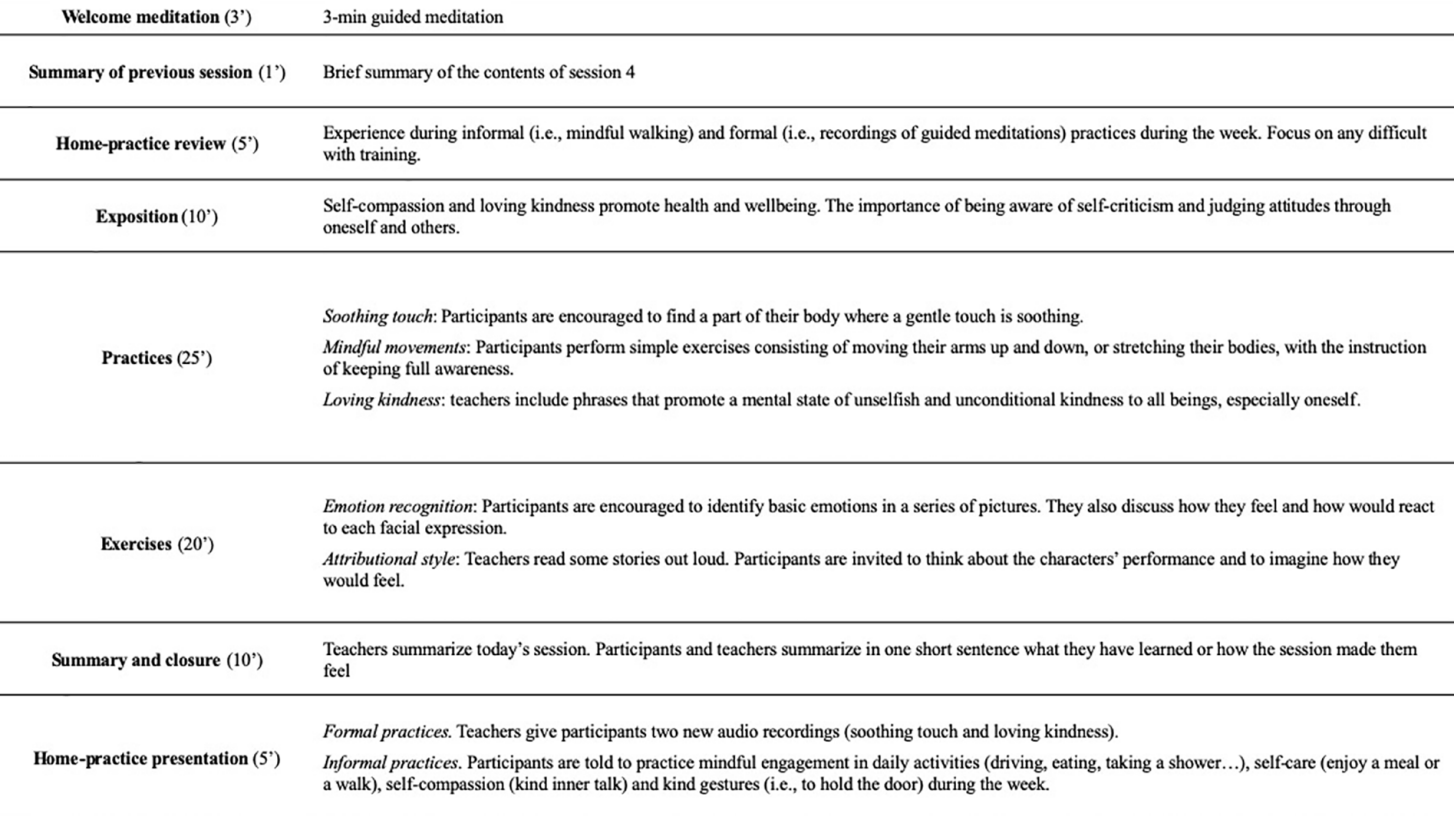
Contents of SocialMind session 5: Compassion and Friendship with Oneself.

SocialMind is an intervention for people with psychosis designed by the authors and presented here for the first time. It is mindfulness-based social cognition training that highlights the importance of cultivating an acceptance-based, nonjudgmental approach to one’s experience. It incorporates formal meditation practices tailored for people with psychosis and social cognition exercises inspired by SCIT (40). Practices of formal meditation consist of focusing attention on a chosen object either inside (i.e., breathing, weight) or outside (i.e., noises, smells) the body, as well as compassion practices such as loving-kindness or soothing touch meditations. They are inspired in practices of three formal mindfulness programs: the Mindfulness-Based Stress Reduction program (MBSR) (18), mindfulness-based cognitive therapy (MBCT) (42), and the Mindful Self-Compassion program (MSC) (43). SocialMind teachers were also certified teachers of these programs, and recommendations of mindfulness programs for psychosis were considered (19, 44). Social cognition training includes attributional bias exercises, mentalizing abilities (i.e., theory of mind), or emotion perception and social cues tasks. The full intervention consists of eight weekly sessions, followed by four fortnightly sessions and five monthly sessions. Each lasts 90 min, and groups are made up of a maximum of 15 participants. For the present study, only weekly sessions were performed (for an example of one of these sessions, see **Figure 1**). One morning and one evening training were scheduled, so participants could choose according to their preferences. The principal teacher (AM) led both groups, with one co-therapist in the mornings (BR) and one in the evenings (AP).

### Statistical Analysis

According to some authors, null-hypothesis contrasts are not necessary in feasibility trials, as they are not designed to test effectiveness of a certain intervention (45, 46). Nonetheless, this trial needs to prove that SocialMind is safe and well tolerated by people with psychosis, and not being able to establish statistical inferences has also been reported as a limitation in feasibility trials (39, 47).

In order to detect moderate-to-large effect sizes (Cohen’s d = 0.7) in indicators of adverse effects with a 95% of probability (1 – ß = 0.95) and a type I error of 5% (α = 0.05), a sample of N = 25 is needed to conduct nonsuperiority tests. First, chi-squared and Mann–Whitney tests were performed to check for equivalence of baselines in morning and evening trainings. Then, the Kolmogorov–Smirnov test revealed if there was any non-normal distribution across main variables. To calculate pre–post differences, a paired-sample one-tailed t-test was carried out when the sample followed a normal distribution and there were no outliers within the dependent variable; for non-normal distributions, the one-tailed Wilcoxon signed rank test for matched pairs was used. Data were analyzed with IBM^®^ SPSS^®^ Statistics Version 21.

## Results

Twenty-seven participants signed informed consent and completed pretreatment assessment. Two of them found a job before groups were formed. Characteristics of the remaining 25 participants are shown in **Table 1**. There were no differences between morning and evening training in age, gender, educational level, job status, and symptoms (SCL-90-R, PANSS, DES-II, STAI-S). Scores on PSP social activities, Z = –2.622, *p* < 0.010, were better among participants of the morning sessions. All interval variables were normally distributed, except for PSP self-care in pretreatment (D = 1.390, *p* < 0.042) and post-treatment (D = 1.605, *p* < 0.012) and PSP aggressive behavior at 8 weeks (D = 1.605, *p* < 0.012). PSP-T had a mean of 57.40 (SD = 14.58) in pretreatment and a mean of 57.44 (SD = 10.72) in post-treatment, and its distribution was normal at both time points.

**Table 1:** Characteristics of the participants (N = 25).

	N (%)/M (SD)
Age in years	45 (9.38)
Gender	
Women	11 (44%)
Men	14 (56%)
Educational level	
Primary (6–9 years)	3 (12%)
Secondary (10–14 years)	18 (72%)
University (>15 years)	4 (16%)
Job status	
Employed	6 (24%)
Unemployed	7 (28%)
Disability insurance	11 (44%)
Student	1 (4%)
Marital status	
Single	20 (80%)
Married	5 (20%)
Duration of illness in years	18.4 (10.9)
Number of hospitalizations	3.04 (3.21)
DSM-5 diagnosis	
Schizophrenia (295.90)	23 (92%)
Schizoaffective disorder (295.70)	1 (4%)
Schizophrenia spectrum disorder not otherwise specified (298.9)	1 (4%)
PANSS score	
Positive syndrome (P)	12.28 (3.21)
Negative syndrome (N)	16.28 (5.20)
General psychopathology (GP)	26.12 (6.04)

PANSS, Positive and Negative Syndrome Scale for schizophrenia; DSM-5, Diagnostic and Statistical Manual of Mental Disorders, 5th Edition.

### Feasibility

**Figure 2:**
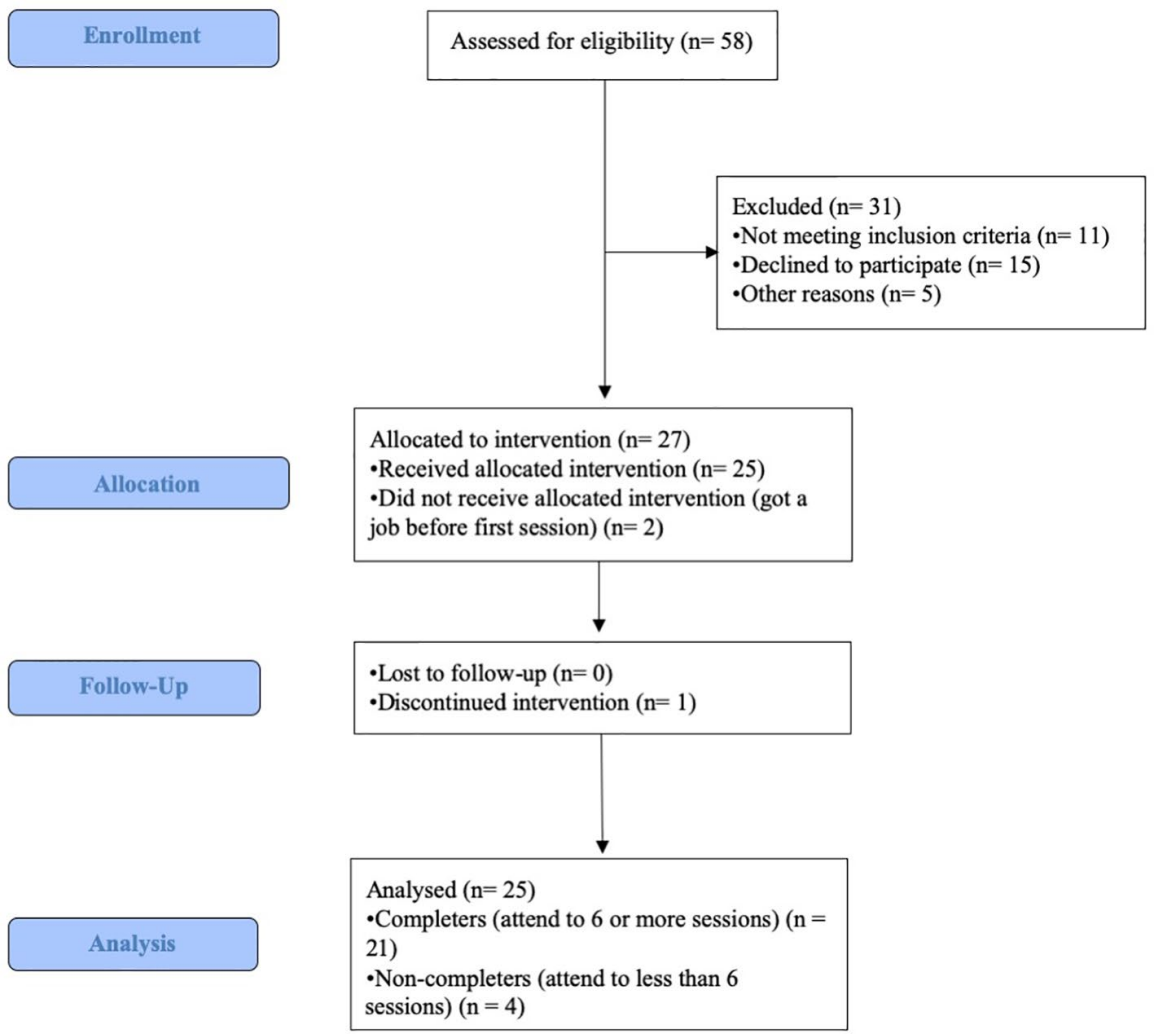
CONSORT flowchart.

The flowchart is shown in **Figure 2**. Fifteen participants were allocated to the morning intervention, and 10 participants were allocated to the evening intervention. The attrition rate was 15%. The remaining participants attended six or more sessions and therefore completed the intensive part of SocialMind training. CSQ-8 scores reveal a median satisfaction of 3 out of 4 points.

### Tolerability and Adverse Effects

None of the participants were hospitalized, went to the emergency room, or asked for an unscheduled appointment with his or her psychiatrist during the 8 weeks of training. After session number 4, one participant stopped coming to the training. He said he was not obtaining any benefit from the intervention. We did not find any indicator of adverse effects in his post-treatment assessment.

**Table 2** shows general symptomatology (SCL-90-R, PANSS), within-session state anxiety (STAI-S), and dissociative experiences (DES-II) before and after SocialMind training. There was no significant increase in symptoms in any of these indicators; however, one participant reported a 3-point (4%) increase in STAI-S after session number 5.

**Table 2:** Pre-post differences (Student’s t-test) on indicators of adverse effects (higher scores in “mean pre” and “mean post” indicates higher presence of symptoms).

Domain	Mean pre	Mean post	Mean difference (SD)	95% one-sided CI
SCL-90-R				
Somatization	0.81	0.72	0.10 (0.55)	[−0.09, ∞)
Obsession–compulsion	1.22	1.03	0.19 (0.62)	[−0.02, ∞)
Interpersonal sensitivity	1.27	1.14	0.13 (0.49)	[−0.04, ∞)
Depression	1.11	0.87	0.24 (0.51)	[0.06, ∞)
Anxiety	1.10	0.99	0.10 (0.44)	[−0.05, ∞)
Hostility	1.03	0.97	0.06 (0.41)	[−0.08, ∞)
Phobic anxiety	1.12	0.97	0.14 (0.47)	[−0.02, ∞)
Suspiciousness	0.97	0.98	0.00 (0.46)	[−0.16, ∞)
Psychoticism	1.11	0.98	0.12 (0.45)	[−0.03, ∞)
PANSS				
Positive syndrome	12.28	11.36	0.92 (3.15)	[−0.16, ∞)
Negative syndrome	16.28	15.48	0.80 (4.49)	[−0.74, ∞)
General psychopathology	26.12	25.88	0.24 (7.60)	[−2,36, ∞)
Dissociative symptoms (DES-II)	9.89	10.80	−0.90 (9.34)^ns^	[−4.10, ∞)
State anxiety (STAI-S)	22.00	16.26	5.74 (5.68)	[3.75, ∞)

SD, standard deviation; CI, confidence interval; SCL-90-R, Symptom Checklist 90—Revised; PANSS, Positive and Negative Syndrome Scale; DES-II, Dissociative Experiences Scale II; STAI-S, State-Trait Anxiety Inventory—State.

^ns^, nonsignificant (p = 0.317).

### Teachers’ Competence and Adherence to SocialMind Manual

Scores on MBI:TAC ranged from 5 (“competent”) to 6 (“proficient”) points, which means that teachers present only minor problems and/or inconsistencies. Checklist scores ranged from 1 to 2 points, with a median value of 2 (“full compliance”) and a mean value of 1.90 (SD = 0.31).

## Discussion

This study explores a new person-centered, group mindfulness-based social cognition training (SocialMind) developed for people with SSDs. We performed a feasibility trial with two goals: to evaluate acceptability and tolerability of SocialMind training and to confirm that a RCT can be implemented.

We did not find any increase in psychotic symptoms, nonpsychotic symptoms, dissociative experiences, hospitalization rates, or functional impairment after SocialMind intervention. Most participants completed the training, and the attrition rate was low compared to previous reports (48). This can be considered an indicator of satisfaction with the intervention, which is also supported by scores on the Client Satisfaction Questionnaire. Recent reviews emphasize that adverse effects may be reported mainly during silence retreats or intensive meditation trainings, and in the context of programs that have not been adapted to people with psychosis (19, 20). SocialMind teachers are psychiatrists certified as mindfulness teachers who have experience with severe mental disorders, which is in keeping with Chadwick’s (44) recommendations. Meditation practices have been specifically adapted for people with SSDs: they are shorter than usual, with simple and concrete instructions, limited periods of silence, and interpersonal practices introduced progressively.

In a systematic review, Aust and Bradshaw (49) identified 11 RCTs of interventions for people with SSDs that explicitly incorporated mindfulness elements. Only five of them were mindfulness-based group interventions. Since then, results from another RCT with over 100 participants have been reported (50). Except for Chadwick et al. and Chien and Thompson (51), sample sizes range from 21 to 44 participants. Positive and negative symptoms are the most frequent primary outcomes among these trials; however, a decrease in symptoms does not necessary improve social function (10). Only one study explored changes in general function against an active comparator, and the authors reported a low effect size (51). Interventions need to improve real-life outcomes (5), and it is desirable for this improvement to be at least as significant as with other comparable interventions; therefore, our RCT will compare the effects of SocialMind training over social functioning against an active comparator. The final score of the Personal and Social Performance (PSP) scale will be our primary outcome measure, which is normally distributed and with values similar to the Spanish adaptation in this trial (36). It is not a self-reported measure, so we also expect that social desirability will be kept as low as possible (52).

It is impossible to assert that an RCT can actually be implemented, because participants were not randomized in this feasibility trial; however, recruitment and adherence rates confirm that people with SSDs in our catchment area are interested in enrolling in a clinical trial. Even though it is possible that participants are retained due to certain elements of SocialMind training, it is more likely that they attended simply because an active intervention was offered to them (53). Therefore, similar results of recruitment and adherence can be expected from an RCT with two active intervention arms (SocialMind *versus* active comparator). If rates are similar, two groups of 10 to 12 people who regularly attend sessions would be formed for each randomization. Effectiveness cannot be explored in a noncontrolled trial, because it is impossible to determine if a change in the outcome variable is caused by the treatment; nonetheless, the purpose of a feasibility trial is not to test effectiveness, so a control group—although desirable—is not necessary. If there had been any major adverse effects, such as visits to the emergency room, hospitalizations, or suicide attempts, either the research team or participants’ psychiatrists would have detected them. Similarly, moderate-to-large increments in psychotic symptoms, anxiety and depressive symptoms, or dissociative experiences would have been detected in 96% of cases (24 out of 25 participants). Because anxiety symptoms are one of the most frequently reported adverse effects of MBIs in psychosis (19), they were monitored thoroughly, and participants registered their anxiety levels before and after each SocialMind session.

SocialMind does not cause any harm to the participants, and descriptive data even suggest that it may have beneficial effects for anxiety and depressive symptoms. A further RCT needs to address this and should explore if there are any improvements in social functioning. SocialMind is also the first mindfulness-based social cognition training, so several social cognition tasks will complete the assessment protocol to explore their role as moderate variables. In order to maximize the therapeutic potential of the RCT, at least two enhancements can be implemented. First, social cognition and functioning are often affected starting in the early stages of psychosis (8), so it might be optimal to apply this intervention as soon as possible. Second, an extended period of booster sessions may set some learning and help to incorporate formal and informal practices as a habit. Our RCT (ClinicalTrials.gov identifier: NCT03309475) will compare the effectiveness of extended SocialMind training (eight weekly sessions, four fortnightly sessions, and five monthly sessions) versus a psychoeducational multicomponent intervention in people between 16 and 40 years of age who have suffered their first psychotic episode within the last five years. We expect that this early, person-centered, group intervention will improve disturbing symptoms, negative emotions, social function, and quality of life, preventing further psychotic episodes and hospitalizations.

## Ethics Statement

This study was carried out in accordance with the recommendations of La Paz University Hospital Research Ethics Committee (PI-3066) with written informed consent from all subjects. All subjects gave written informed consent in accordance with the Declaration of Helsinki. The protocol was approved by La Paz University Hospital Research Ethics Committee and registered under ClinicalTrials.gov identifier NCT03434405.

## Author Contributions

RM designed the investigation, collected and analyzed the data, and wrote the final version of the manuscript. AM-S designed the intervention, led SocialMind groups, and contributed to the final version of the manuscript. BR-V, CB, and AP developed the intervention, were teachers in SocialMind groups, and contributed to the final version of the manuscript. GL developed the intervention and contributed to the final version of the manuscript. MB monitored the process and contributed to the final version of the manuscript. All the authors read and approved the final version of the manuscript.

## Funding

The research was funded by the European Regional Development Fund (ERDF) and the Institute of Health Carlos III (ISCIII) (grant number PI 17/00768).

## Conflicts of Interest Statement

The authors declare that the research was conducted in the absence of any commercial or financial relationships that could be construed as a potential conflict of interest.
